# Untangling behaviours: independent expressions of female–female aggression and snake-like hissing in the blue tit (*Cyanistes caeruleus*)

**DOI:** 10.1038/s41598-023-43652-3

**Published:** 2023-09-28

**Authors:** Robin van Iersel, Gust Boiten, Rianne Pinxten, Marcel Eens

**Affiliations:** 1 Department of Biology, Behavioural Ecology and Ecophysiology Group, University of Antwerp, Universiteitsplein 1, 2610 Antwerp, Belgium; 2 Research Group Didactica, Antwerp School of Education, University of Antwerp, 2610 Antwerp, Belgium

**Keywords:** Animal behaviour, Behavioural ecology

## Abstract

Aggression plays a crucial role in deterring predators and securing resources to promote fitness. Nevertheless, studies focussing on female aggression remain scarce. In songbirds, aggression is prevalent during the breeding season, when same-sex individuals compete for limited resources. Additionally, females of some bird species exhibit snake-like hissing behaviour during incubation presumably to lower predation rates and improve fitness. Such behaviours may co-vary, forming a behavioural syndrome that could constrain trait expression. Here, we investigated a resident population of blue tits (*Cyanistes caeruleus*), to examine the repeatability and covariation of female–female aggression and hissing behaviour, aiming to determine if these constitute a behavioural syndrome. We quantified female–female aggression during simulated territorial intrusions and measured number of hissing calls in response to a simulated predator intrusion into the nest box. We found that both female–female aggression and hissing behaviour were repeatable traits, and that older females approached the intruder less. However, we found no evidence of covariation between female–female aggression and hissing behaviour. Thus, our findings suggest that female–female aggression and hissing behaviour, although both displayed in a nest defence context, are evolutionarily independent traits in the blue tit.

## Introduction

Aggressive behaviour in nature is prevalent, yet often perceived as a predominantly male trait^1–3^. By displaying aggression individuals are able to secure resources that promote fitness, such as food, territories and mates, and are able to deter predators^4, 5^. However, while studies focussing on male aggression are common, fewer studies have tried to understand the causes and consequences of female aggression^1, 6, 7^. This may in part be attributable to female aggressive displays being at times less conspicuous, or that the study of aggression is often limited to the paradigm of sexual selection^3^. Yet, increasingly evidence suggests that the mechanisms driving aggression may be sex dependent^8, 9^. Aggressive behaviour in males is primarily driven by mate choice and intrasexual competition for mating opportunities (i.e. sexual selection), while female aggressive behaviour may be under different selection pressures, more often related to mediating access to ecological resources^9^. Consequently, what drives female aggression, and how this may differ from males, is poorly understood^3, 10^.

In songbirds, intrasexual aggression is often particularly severe because individuals of the same sex require the same limited resources to maximise reproductive success^3^. Female–female aggression during the breeding season may serve to prevent intruding females from settling nearby, which could reduce food competition, predation risk and mate competition^11^. Additionally, female–female aggression may allow females to protect their nest sites from destruction, nest parasitism or take-overs^7, 11–13^. Hence, intrasexual aggression may enhance fitness by promoting resource acquisition and reducing competition, and may be an important predictor of reproductive success^3, 13, 14^. However, while increased intrasexual aggression may provide crucial benefits, there may also be severe trade-offs, as aggression may be costly in terms of energy expenditure or risk of injury^15^. To mitigate such costs, individuals often mediate intrasexual aggressive interactions via aggressive signalling, such as calls^16^. Overall, intrasexual aggression in songbirds is an important strategy for competing over limited resources and protecting nest sites, but may also carry significant costs, highlighting the need to further study the adaptive significance of intrasexual aggression.

Female aggression is not restricted to an intraspecific context, but may also be employed to scare off potential predators in a nest defence context. When a predator approaches a nest site, some bird species, such as blue tits (*Cyanistes caeruleus*), great tits (*Parus major*), and black-capped chickadees (*Poecile atricapillus*), produce loud broadband hissing calls in an attempt to deter the predator^17^. This hissing behaviour, in which females perform a kind of hiss while fluttering their wings, is presumed to be a case of mimicry where birds emulate the hiss of a snake to scare off predators^5, 18^. Indeed, such behaviours, while risky and not always effective, may deter certain predators as a last line of defence and increase survival, although they may also incur a reproductive cost (i.e. reduced egg production, fledging success or fledgling quality)^5, 19^. Individuals vary in the degree in which they hiss and whether they hiss, as not all individuals show such hissing behaviour in the presence of a predator, instead either hiding in the nest or not covering the eggs, lowering their own mortality risk but increasing the predation risks of the offspring^6, 20^.

Consistent (i.e. repeatable) between-individual variation in behaviour is commonly referred to as a personality trait^21, 22^. Several studies have shown that intrasexual aggression and hissing are repeatable behaviours, with evidence of consistent individual variation in intrasexual aggression observed in various taxa, including a number of songbirds (reviewed in Salazar et al.^23^). While fewer studies have focussed on hissing behaviour, it has also been found to be individually consistent in female great tits, highlighting the recognition of these traits as aspects of animal personality^5, 19^. Understanding the adaptive significance of consistent individual variation in animal personality traits remains a major challenge for evolutionary ecologists, as behavioural plasticity is theoretically expected to provide a selective advantage^24^. Suites of functionally distinct personalities may be correlated with one another resulting in what we call behavioural syndromes^25^. In line with the constraint hypothesis, behavioural syndromes may arise due to underlying proximate links (e.g. hormones, genes) that regulate expression of multiple behaviours, which may impose constraints on plasticity^26^. As such, individuals which are aggressive in a conspecific context may also be aggressive when confronted with predators, but what may be adaptive in one context may be maladaptive in another^25, 27^. For example, aggression may be suitable in a context where individuals compete for food or mates, but if those same individuals are needlessly aggressive in a predator context, they may be exposed to unnecessary risk^25^. Conversely, in male western bluebirds (*Sialia mexicana*) high nest defence is expected to promote reproductive success, but is related to high male-male aggression (coupled with reduced parental care), resulting in fewer fledged offspring^28^. Alternatively, behavioural syndromes may arise as adaptive responses to selection pressures^26, 29^. Regardless, the presence of behavioural syndromes suggests that behavioural traits are not evolutionarily independent, and may hence be key to our understanding of how behavioural traits evolved and are maintained, and why individuals at times display suboptimal behaviour^25, 29^. Indeed, recent evidence shows that behavioural syndromes have the potential to constrain short term adaptive evolution, and may determine fitness that can be achieved across different environments^30, 31^.

Aggression and hissing behaviour are both risk-taking but functionally distinct behaviours, yet both serve as deterrents to potential threats while nesting. Although these behaviours may be correlated, studies linking aggression with other risk-taking behaviours (e.g. activity in a novel environment, approaching novel objects) have yielded mixed results, where contextual overlap may enhance likelihood of finding correlations due to the experimental environment affecting measured behaviours in the same way (reviewed in Garamszegi et al.^32^). Few studies have tried to link aggression across a conspecific and predator context^23^, particularly in females (but see Thys, Pinxten, et al.^19^ and Cain et al.^27^). In female dark-eyed juncos (*Junco hyemalis*), relative levels of aggression towards predators, same- and opposite-sex intruders were found to be correlated, suggesting a potential link between conspecific aggression and anti-predator behaviour^27^. However, previous research in female great tits did not find evidence of a behavioural syndrome between aggression and hissing^19^, clearly indicating differences between species in the relationship between aggression and risk-taking behaviours. Furthermore, behavioural syndromes may be sex-specific, as illustrated by a study in blue tits where nestling defence and handling aggression are associated in females but not males^6^. These findings demonstrate the complexity of behavioural interactions and highlight the need for further investigation into the potential interplay between aggression and hissing in relevant species.

In this study, we aim to assess the consistency of female–female aggression and hissing behaviour, and explore their potential relationship to investigate the presence of a behavioural syndrome, using the blue tit as our model organism. To this end, we will perform simulated territorial intrusions, in which a caged taxidermy mount is placed at the nest site during the breeding season, to measure female–female aggression. Furthermore, we will introduce a head model of a common nest predator (Great spotted woodpecker, *Dendrocopos major*) into the nest box entrance to measure hissing behaviour. The blue tit, a small and aggressive cavity nesting species^33, 34^, exhibits a wide range of individual variation in aggression, making it an excellent model organism for this study^35–37^. While blue tits are primarily socially monogamous, they possess a facultative polygynous mating system with frequent instances of extra-pair paternity^34^. Intra-sexual aggression among female blue tits plays a crucial role in preventing male polygyny, as failure to do so results in reduced parental care and decreased survival chances for the primary female^36^. These characteristics make the blue tit an exceptional species for investigating the interplay between aggression and hissing behaviour.

## Methods

### Study site and population

This study was conducted using a resident suburban population of free-living blue tits at Wilrijk, Belgium (51°09′46″N, 4°24′13″ E, see Sun et al.^38^). The study area contains 61 nest boxes only available for blue tits by way of a small entrance size, barring entrance to most other bird species. In addition, some blue tits nested in nest boxes provided for a resident population of great tits. Nest boxes were located ± 2 m of the ground, and were monitored daily from the start of the breeding season (mid-March 2022), to follow nest development, egg-laying and incubation, and detect potential laying interruptions^39^. To aid individual recognition, individuals were fitted with colour rings, an aluminium ring and tagged with a Passive Integrated Transponder (PIT tag, EM4102, Eccel Technology Ltd., Great Britain), as nestlings or as adults, during routine population checks in winter and the breeding season since 2015. In addition, sex and age class (yearling or older) were determined for each individual.

### Simulated territorial intrusions

In order to measure intra-sexual aggression, we determined the behavioural response of 38 female blue tits when confronted with a simulated female territorial intrusion near their nest box. Simulated territorial intrusions were performed twice, on day 3 and 7 during the egg-laying period, although some tests were delayed to account for laying interruptions (Testing day; mean ± SD is 3.2 ± 0.4 and 7.2 ± 1.1 for day 3 and 7 respectively; see Boiten et al.^35^). To simulate intrusions, one of three female blue tit taxidermy mounts (decoys) were used, protected by a small mesh cage (15.5 × 15.5 × 17.5 cm). Prior to the experiment, a camera (Sony HDR-XR550VE or JVC GZ-R405BE) was placed 5–7 m from the nest box. Then a decoy was placed on top of the nest box at a random outward facing angle, and an observer took place behind the camera. Once the resident female arrived and entered a radius of 15 m around the nest box, she was tested for 5 min. If a bird did not show up within 15 min, another attempt was made at least 1 h later, or the next day. On 12 occasions tests failed due to non-arrival of an individual and were rescheduled for the next day. Ultimately, 3 birds had to be excluded as they never showed up even after repeated attempts. Relevant behaviours displayed outside of the camera frame (± 1.5 × 2.7 m) were described by the observer, so that these could be quantified when analysing the recording. During trials, an observer noted colour ring combinations for later validation of the individual’s identity. One of three observers and three decoys were randomly assigned per trial. The simulated territorial intrusion experiments were conducted in March and April 2022, and were performed between 08:00 and 13:30.

Camera footage was analysed using ‘Behavioral Observation Research Interactive Software’^40^. The following behaviours were scored: minimum approach distance to the decoy (0 m when perched on the decoy, 0.01 m when inside the nest box, estimated to 0.1 m accuracy when closer than a meter and 1 m accuracy further away), number of pecks on the decoy, amount of time spent on the decoy, in the nest box, and in front of the nest box entrance (in seconds) and number of calls. If an individual was only tested once, the data was discarded, resulting in a total of 76 aggression tests at 38 nest boxes.

As a previous study already showed that blue tits respond aggressively towards taxidermy mounts^35^, we did not test whether the reaction to the decoy was the result of neophobia (e.g. using an empty cage). Blue tits respond significantly more to a wooden platform with a clay blue tit model perched on top, than to only the wooden platform^41^. Moreover, previous studies on house sparrows (*Passer domesticus*) and eastern bluebirds (*Sialia sialis*) have shown that simulated territorial intrusions using empty cages do not evoke an aggressive response^42, 43^, as such the use of empty cages to test for neophobia was deemed unnecessary.

### Hissing

In order to quantify the hissing response, we performed artificial predator intrusions (hereafter ‘hissing tests’; Grunst et al.^44^; Krams et al.^5^; Thys et al.^7^), using either a yellow marker, one of two 3D-printed woodpecker head models, or both (6.3, 20.8 and 72.9% of females respectively). A yellow marker was initially used as the woodpecker models were not yet available. Therefore, we also validated whether the object used had an impact on hissing (See “Hissing behaviour” described below). The woodpecker heads were modelled after the great spotted woodpecker (*Dendrocopos major*), and subsequently scaled so they would fit through the nest box entrance (Supplementary Fig. S1). The object was entered ± 3 cm into the nest box entrance for one minute. During this time, the number of times a female hissed was recorded using a portable audio recorder (TASCAM DR-07MKII). Following the trial, the presence and identity of a female was confirmed by scanning PIT-tagged individuals (using LID575 Midrange Reader) or opening the nest box. If a female was not present during a hissing test, the test was attempted later that day or the next day. Hissing behaviour was measured on the 3rd, 7th and 9th day of incubation between 08:30 and 17:00, by one of three observers. Due to the absence of birds during multiple attempts, some birds were only tested twice (17 females) and one bird was only tested once, whereas all other birds (30 females) were tested three times. In total this resulted in 125 hissing tests being performed, using 48 females. Observers and marker or woodpecker models were randomly assigned per trial.

### Statistical analysis

All statistical analyses were performed in R (v4.3.0)^45^. Results with a *p* value < 0.05, or 95% confidence intervals that do not overlap with zero are considered significant.

#### Dual multiple factor analysis

We used the *FactoMineR* package (v2.6)^46^ to perform a Dual Multiple Factor Analysis (DMFA), with varimax rotation, on the behaviours measured during the aggression test for dimensionality reduction. DMFA was used to account for the repeated measures structure in the data, where behavioural parameters measured in the first and second trial were split into two separate data tables^47, 48^. Prior to the DMFA, the separate tables were z-standardized, and it was validated whether there was redundancy between behavioural parameters and sampling adequacy by performing Bartlett’s Test of Sphericity (χ^2^ = 170, *p* < 0.001) and the Kaiser–Meyer–Olkin test (KMO = 0.52) respectively (*psych* v2.2.5)^49, 50^. To aid interpretation, approach distance was multiplied by − 1 prior to analysis so high values indicate a more aggressive response^51^. Factors were extracted based on Horn’s parallel analysis (*paran* v1.5.2)^52^. Loadings were calculated by dividing a factor’s coordinates by the square root of that factor’s eigenvalue. Factor loadings > 0.35 were considered to significantly contribute to the construction of the factors.

#### Aggression and hissing

Using the factors created by the DMFA as a proxy for aggression, we then calculated adjusted repeatabilities (using a separate univariate model for each factor), by dividing the between-group variance by the total (between and within-group) variance^53^. To do so, we used the *rptR* package (v0.9.22), and determined repeatabilities based on 1000 bootstraps simulations^54^. Significance was determined via likelihood ratio tests. Female ID was included as a random effect. To calculate adjusted repeatabilities, age (as a two-level factor, 0: yearling, 1: older), clutch size, Julian date (relative to the 1st of April), time (relative to sunrise), observer ID and decoy ID were included as fixed effects. Clutch size, Julian date and time were each standardized to aid interpretation. Age was unknown for two individuals, so these were discarded from the analysis (i.e. N = 72, at 36 nest boxes). Additionally, identical models were run for each independent behavioural trait rather than each DMFA factor, to assess the repeatability of each trait independently and to provide data for eventual future meta-analysis. For the latter analyses, approach distance and calls were excluded as the data did not have enough variation to be accurately described by any distribution. Models for the number of pecks and time in entrance were fit using a Poisson distribution. All other models were fit using a Gaussian distribution.

Adjusted repeatability of hissing was determined similarly to aggression. All females, including those tested fewer than three times, were included in the analysis. Female ID was included as a random effect. To calculate adjusted repeatability, age (as a two-level factor, 0: yearling, 1: older), Julian date (relative to the 1st of April), time (relative to sunrise), day of incubation, observer ID and object ID (woodpecker 1, woodpecker 2, or yellow marker) were included as fixed effects. Incubation day, Julian date and time were each standardized to aid interpretation. The model was fitted using Poisson errors and 1000 bootstrap simulations. For both aggression and hissing, bootstrap confidence intervals of fixed effects were determined based on 500 simulations, using the lme4 package (v1.1-30)^55^.

Finally, we test for a behavioural syndrome between aggression and hissing using Spearman’s rank correlation coefficient. We used the average scores of the first and second aggression tests (i.e. scores obtained from the DMFA and the individual aggression parameters) as well as the average scores of the first and second hissing tests (i.e. phenotypic correlation of individual means)^56^, which were available for 37 females.

### Ethical note

This study was approved by the ethical committee of the University of Antwerp (ID number: 2022-25) and was performed in accordance with Belgian and Flemish laws regarding animal welfare, adhered to the ASAB/ABS guidelines for the use of animals in behavioural research and teaching, and complied with ARRIVE guidelines. The Royal Belgian Institute of Natural Sciences (KBIN) provided ringing licenses for all authors and technicians. Handling time was minimized as much as possible. All other methods described above are non-invasive. Few nests were deserted in the experimental year (2022), and the desertion rate remained within the expected rate under undisturbed conditions in our population (unpublished data).

## Results

### Female–female aggression

Most females (89.5%) landed on the decoy at least once, resulting in little variation in approach distance (mean ± SD; 0.15 m ± 0.72, range 0–5 m). Similarly, a small proportion of females (28.9%) produced calls during at least one of the two trials, showing little variation in the number of calls (mean ± SD; 1.17 ± 4.51, range 0–33). By contrast, time on decoy (mean ± SD; 93.30 s ± 72.78, range 0–264 s), number of pecks (mean ± SD; 15.42 ± 18.75, range 0–82), time in nest box (mean ± SD; 114.51s ± 108.32, range 0–300) and time in entrance (mean ± SD; 16.34 s ± 25.69, 0–162) during the 5 min observation period all varied substantially. The majority of females (89.5%) pecked the decoy, spent time on the decoy (97.4%), in the nest box (81.6%), or at nest box entrance (73.7%) at least once across the two trials.

The DMFA resulted in two factors being extracted, each factor explaining 42% and 24% of variation respectively. Individuals with a high score for the first factor spent more time on the decoy, pecked the decoy more, and also spent more time in the nest box entrance, while individuals with a lower score spent more time in the nest box (Table 1). Individuals with a high score for the second factor approached the decoy more closely, and spent more time in the nest box. Conversely, individuals with a lower score called more. The second factor was primarily influenced by variation caused by a small number of individuals, who either called or did not land on the decoy during a limited number of trials (14 and 5 respectively).

**Table 1:** DMFA loadings, including each factor’s eigenvalue as well as percentage of explained variance.

	Factor 1	Factor 2
Eigenvalue	2.55	1.43
% Variance explained	42.44%	23.86%
Approach distance	0.138	**0.662**
Time on decoy	**0.576**	0.054
Number of pecks	**0.514**	0.087
Time in nest box	**− 0.484**	**0.418**
Time in entrance	**0.387**	0.069
Number of calls	− 0.014	**− 0.609**

Data includes 38 individuals each measured twice. All loadings greater than 0.35 are marked in bold and considered significant.

Both the first (R [95% CI] = 0.34 [0.09, 0.70], *p* = 0.037) and second factor (R [95% CI] = 0.83 [0.75, 0.94], *p* < 0.001) were significantly repeatable. Older females had lower overall scores for the second factor than yearlings, but age did not affect the first factor scores (Table 2). Clutch size, Julian date, decoy ID, start time and observer ID did not have an effect on either factor (Table 2). Considering the individual aggression parameters, all four were also repeatable (Table 3). Older females spent less time in the nest box, whereas individuals that had larger clutches or early nests spent more time perched in the nest box entrance (Table S1).

**Table 2:** Univariate mixed models for repeatability of factor scores.

	Factor 1	Factor 2
Fixed effects	β [CI]	β [CI]
Intercept	− 0.50 [− 1.58, 0.46]	0.35 [− 0.29, 0.96]
Age—Older	0.80 [− 0.02, 1.72]	**− 0.77 [− 1.56, − 0.04]**
Clutch size	0.11 [− 0.25, 0.48]	− 0.03 [− 0.24, 0.19]
Julian Date	− 0.17 [− 0.65, 0.30]	0.06 [− 0.37, 0.52]
Decoy B	0.27 [− 0.65, 1.27]	− 0.13 [− 0.44, 0.30]
Decoy C	− 0.17 [− 1.09, 0.81]	− 0.02 [− 0.47, 0.40]
Start time	0.07 [− 0.32, 0.47]	0.08 [− 0.10, 0.25]
Observer B	0.12 [− 0.94, 1.18]	0.21 [− 0.22, 0.68]
Observer C	0.11 [− 0.82, 1.01]	0.09 [− 0.39, 0.55]

Given are fixed effect estimates and confidence intervals. Fixed effects which do not overlap with zero (95% CI) are in bold.

**Table 3:** Repeatabilities of individual aggression parameters.

	Adjusted repeatability	*p* value
Time on decoy	0.484 [0.276, 0.777]	0.00427
Number of pecks	0.539 [0.159, 0.803]	0.00591
Time in nest box	0.416 [0.191, 0.732]	0.00619
Time in entrance	0.717 [0.392, 0.919]	0.000269

95% confidence intervals are given in brackets. Repeatabilities are given on link-scale where applicable. For fixed effects estimates see supplementary Table S1.

### Hissing behaviour

During the 1 min hissing test, female hissing across all hissing tests ranged from 0 to 70 (mean ± SD; 17.70 ± 18.61), with little variation across the different tests (range 0–69; 0–70; 0–56 and mean ± SD; 14.33 ± 19.27; 21.72 ± 18.01; 16.77 ± 17.87 for the first, second and third hissing tests respectively). Females hissed in 81 out of 125 trials (mean ± SD; 27.31 ± 16.47, N = 81), and the majority of females (77%, N = 48), hissed at least once across all trials. Hissing was highly repeatable (R [95% CI] = 0.72 [0.55, 0.86], *p* < 0.001). Overall, hissing was not affected by age, Julian data, object ID, incubation day and time of day, while the number of hissing calls varied among observers (Table 4).

**Table 4:** Univariate mixed model for repeatability of hissing.

	Temporal
Fixed effects	β [CI]
Intercept	0.72 [− 0.60, 1.92]
Age—Older	− 0.70 [− 2.12, 0.51]
Julian date	0.45 [− 0.36, 1.14]
Object—Woodpecker 2	0.12 [− 0.67, 0.97]
Object—Yellow marker	− 0.69 [− 1.58, 0.37]
Day of incubation	0.40 [− 0.12, 0.91]
Time	0.36 [− 0.07, 0.76]
Observer B	**1.44 [0.53, 2.44]**
Observer C	0.90 [− 0.01, 2.05]

Given are fixed effect estimates and confidence intervals. Fixed effects which do not overlap with zero (95% CI) are in bold.

### Behavioural syndrome

**Figure 1:**
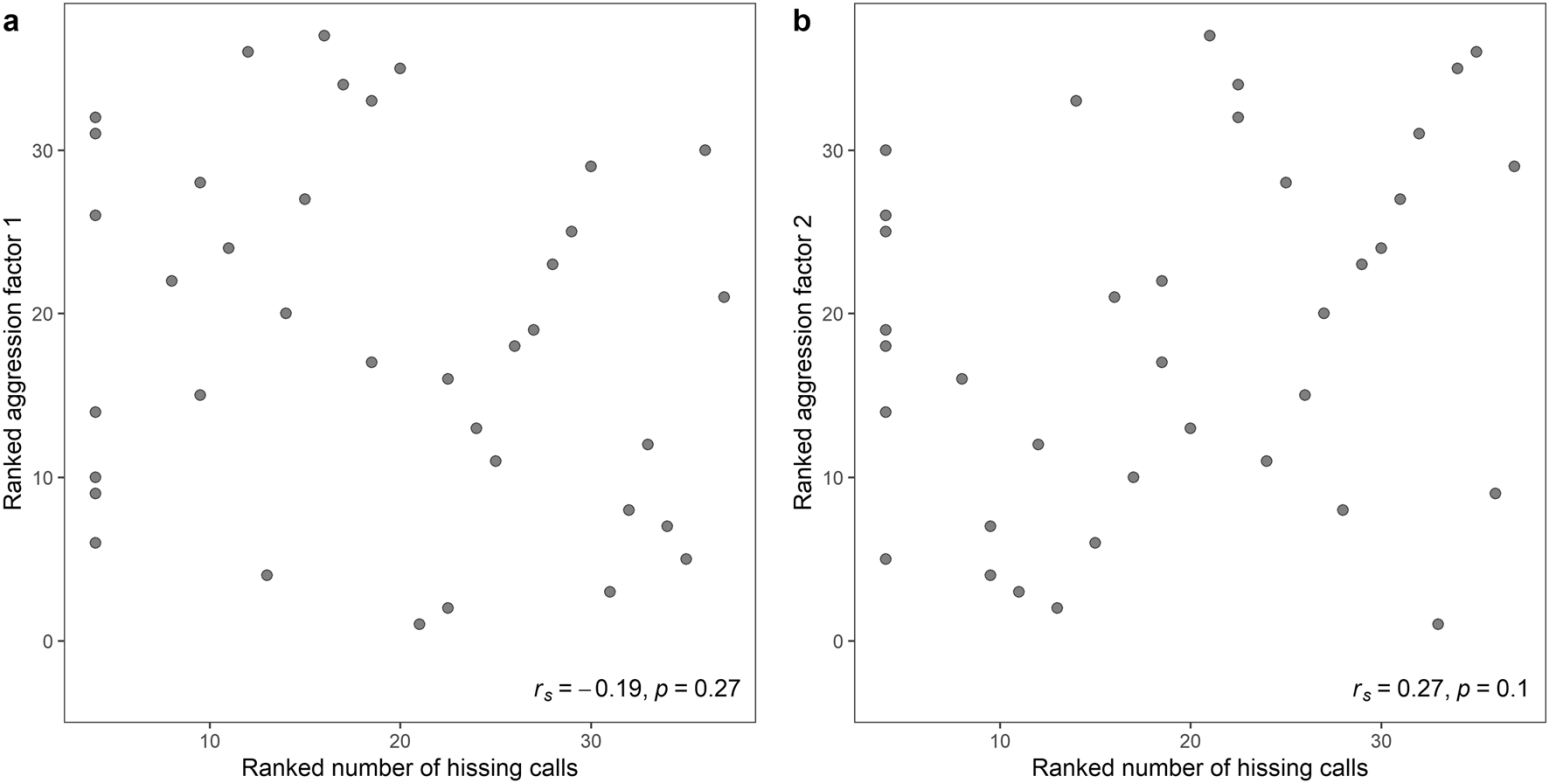
Scatterplot of the phenotypic correlation of individual means between female–female aggression and number of hissing calls, for the first factor (**a**), and the second factor (**b**). Data points are ranked in accordance with the Spearman correlation analysis, with the Spearman correlation coefficient and p-value in the bottom right corner.

There was no significant relationship between hissing and the first (r(35) = − 0.19, *p* = 0.269) and second factor of aggression (r(35) = 0.27, *p* = 0.104; Fig. 1). Similarly, hissing did not correlate with number of pecks (r(35) = − 0.02, *p* = 0.904), time on decoy (r(35) = − 0.02, *p* = 0.892), time in nest box (r(35) = 0.19, *p* = 0.268), time in entrance (r(35) = − 0.28, *p* = 0.094), number of calls (r(35) = − 0.16, *p* = 0.356) or approach distance (r(35) = − 0.00, *p* = 0.995). Out of the 37 females for which we collected both hissing and aggression scores, 7 did not hiss.

## Discussion

In this study, we assessed the repeatability of two nest defence behaviours in female blue tits, specifically female–female aggression and hissing behaviour, and determined if they form a behavioural syndrome to gain insight in potential evolutionary constraints. Our findings reveal that female–female aggression is mildly repeatable while hissing behaviour is highly repeatable, indicating both traits constitute aspects of personality in our resident blue tit population. Finally, we found no evidence that females which are more aggressive during simulated territorial intrusions by a conspecific female produce more hissing calls when a predator enters their nest box.

Females in our population displayed a high level of aggression during the simulated territorial intrusions, with only a small number not landing on the decoy. Those that spent more time sitting on the decoy were more likely to engage in attacks, as is reflected by the first factor extracted through DMFA. As a result, we consider this factor a reliable proxy of female–female aggressiveness. This first factor is mainly characterized by the amount of time spent on the decoy, attacking and sitting in the nest box entrance, while less aggressive individuals spent more time inside the nest box. The second factor is instead characterized by spending more time near the decoy and in the nest box, or alternatively, staying further away and calling in response to the intruder. Individuals varied consistently in their degree of female–female aggression (i.e. the first factor) within the breeding season, with a repeatability that was only moderate (R = 0.34), which is in line with typical repeatability values for behavioural measures^57^. Individuals were highly consistent in whether they approached the decoy and entered the nest box, or stayed at a distance and alarm called (i.e. the second factor, R = 0.83). Similarly, considering individual aggression parameters (time on the decoy, time in nest box, time in entrance, number of pecks), we observed moderate to high levels of repeatability. That aggression in our blue tit population is consistent within individuals is not surprising, as aggression is generally a repeatable trait^32, 57^. Indeed, our results are in line with previous results on aggression in female great tits^7, 19^. Moreover, studies on male aggression during simulated territorial intrusions in another blue tit population reported even higher repeatability (R = 0.56^23^) compared to our observations in females. Yet another study on blue tits found that the repeatability of female nestling defence and handling aggression to be equal or higher, respectively, to that of males^6^, suggesting potential sex and/or population dependent variability in the repeatability of aggression. In our study, we focussed on the repeatability of female aggression during one breeding season and one breeding stage (egg laying period). It is important to note that short inter-test intervals can lead to inflated repeatability estimates due to the likelihood of individuals being of similar physiological and behavioural states^22, 57–59^. While a previous study on female intrasexual aggression in great tits found that short- and long-term repeatabilities are similar^19^, future studies should examine the consistency of female aggression in blue tits across multiple breeding stages and years.

Concerning hissing behaviour, we found that the vast majority of individuals hissed during at least one of the hissing trials (77%), filling a gap in existing literature by quantifying blue tit hissing calls. In blue tits, research on hissing behaviour has been scarce, and studies have used different methods (i.e. reporting on pooled binary data from multiple tit species^60^ or measuring hissing as different behaviours on an interval scale^6^) complicating comparisons among different blue tit populations. The proportion of hissing females in our population is comparable to earlier results found in the great tit^5, 61, 62^. By contrast, blue tits in our population exhibited a higher average number of hissing calls (mean: 17.70, range 0–70, including all individuals) compared to various populations of great tits that underwent similar tests (i.e. a stuffed woodpecker inserted into a nest box for 60 s). Specifically, previous studies by Krams et al.^5^ reported mean values of 2.33 (range 1–25) and 2.42 (range 1–5), excluding non-hissers, across two populations, while Thys et al. ^20^ observed a mean of 12.2 (range 0–43, including all individuals). At present it is unclear why there are such large differences in female hissing behaviour among populations and species, although it has been suggested that differences in the number of hissing calls may, in part, reflect population level differences in the level of predation^5^.

Female blue tits exhibited a high level of consistency in the number of hissing calls, in agreement with previous studies on hissing in great tits^5, 19, 61^. In contrast to great tits, literature on the repeatability of hissing behaviour in blue tits remains limited. In a previous study, female blue tits’ response to the opening of the nest box was measured as different behaviours on an interval scale of 1–4, and consistently showed within-year (i.e. short-term) repeatability over a span of 6 years (ranging from R = 0.22 to R = 0.46) but not long-term repeatability^6^. Another study, which utilized a binary measure of hissing and presented pooled data from multiple tit species, also reported short-term repeatability (R = 0.27)^60^. In our study, we observed much higher short-term repeatability (R = 0.72). This disparity potentially results from our methodology, as we measured hissing by introducing an object into the nest box entrance rather than opening the nest box. Notably, the specific object used during our hissing tests had no discernible effect in our study, suggesting blue tits may respond similarly to any perceived predation attempt. However, the most probable cause for the differences between the previously mentioned studies stems from our use of a continuous scale for measuring hissing calls (i.e. number of hissing calls in 1 min). In doing so we were able to obtain more accurate measures of between and within individual variation, and hence repeatability. Whether the number of hissing calls made is repeatable long-term remains to be studied in the future. The repeatability of hissing behaviour in our study may be partially biased upwards by repeatable differences in the local environment effects (e.g. predation pressure, density, climatic conditions)^63^, although previous research has shown that hissing behaviour in great tits is repeatable both on the short- and long-term with the difference being minimal^19^. It is also worth pointing out that there was a small observer effect (most likely due to one observer having done only a small number of hissing tests), but by calculating adjusted repeatabilities we controlled for any confounding effects this may have caused^53^. Our findings thus provide novel evidence that the number of hissing calls made by female blue tits during hissing tests is a consistent and repeatable trait, complementing previous research on short-term repeatability of hissing behaviour in blue tits.

Although both female–female aggression and hissing behaviour are repeatable, we did not find evidence for a behavioural syndrome. Our results are in line with previous findings on females in the closely related great tit^19^, as well as with a study on male blue tits, showing that male aggression did not covary with risk-taking behaviour^23^. Since our measures of female–female aggressiveness and hissing behaviour were assessed across different breeding stages, it is possible that individuals underwent a change in behaviour, potentially obfuscating the presence of a behavioural syndrome^56^. We also acknowledge that multivariate mixed-effect models are preferred, as they allow for decomposing phenotypic correlations into both between- and within-individual components^64^, but the sample size in our study was too limited to use this approach. We consider the phenotypic correlation of individual means used here as the best proxy for estimating the between-individual correlation, especially given that we have demonstrated repeatability in both traits^56^. Moreover, phenotypic correlations may provide considerable information regarding both the direction and magnitude of the underlying genetic correlations^65^. Hence, based on these considerations, our results provide no evidence for the presence of a behavioural syndrome. Against our expectations, this suggests that selection did not favour a suite of correlated behaviours and that female–female aggression and hissing behaviour evolved independently (i.e. behaviours did not evolutionarily constrain one another). Ultimately this implies that female–female aggression and hissing behaviour are likely not underpinned by the same underlying mechanisms. To validate this conclusion, further research employing more rigorous statistical methods is warranted. Moreover, identifying the genetic, hormonal, maternal or permanent environmental components that regulate both female–female aggression and hissing behaviour would provide a comprehensive understanding of the evolutionary processes that have shaped these behaviours.

Considering factors affecting female behaviour during the simulated territorial intrusions, we found that age was not directly related to female–female aggression (i.e. the first factor), which contrasts previous findings in female great tits^7^ and male blue tits^41^. By contrast, yearling females tended to approach the decoy and nest box more frequently, while older females remained at a distance while calling (i.e. the second factor). This age-related decrease in propensity to approach the decoy, but increase in signalling, may reflect a within-individual decrease in risk-taking behaviour and/or an increase in status signalling. These age-related changes in behaviour may be the result of either senescence^19, 66^, the ability of older individuals to better assess threats^19, 41, 67^, or older individuals needing to invest less in territorial defence than yearlings due to their pre-established territories^19, 67^. Alternatively, aggressive females may selectively disappear from the population due to increased risk-taking behaviour^19^, as previous studies have shown that more shy (and presumably less aggressive) individuals may prioritize survival over reproductive success^68, 69^. However, previous work on female great tits has demonstrated that a similar age-related decrease in aggression with age was the result of within-individual age-related plasticity and not the selective disappearance of more aggressive females^19^. While behaviour during the simulated territorial intrusions varied by age, we found no such effect for the number of hissing calls produced in response to a predator, consistent with findings reported in great tits^19, 20^. Considering the individual aggression parameters, we only found an age-related effect for the time spent in the nest box, with older individuals spending less time in the nest box. This pattern potentially reflects the earlier mentioned decrease in risk-taking behaviour with age. Finally, we found that females with early and large clutches spent more time in front of the nest box entrance. Although the reason for this behaviour is not entirely clear, it is known that female–female aggression is particularly high early in the breeding season as females compete for high-quality nest sites and deter floater females^70, 71^. Furthermore, in many species including the blue tit (and also in our population, where clutch size and laying date are negatively correlated: r(74) = − 0.29, *p* = 0.01, unpublished data), early laying females have a larger clutch size and are of higher quality^72^ and may hence be protecting their clutch more by sitting in the nest box entrance.

In conclusion, both female–female aggression and hissing behaviour were found to be repeatable and therefore likely represent important components of blue tit personality. However, our study found no evidence of a behavioural syndrome, as the two behaviours did not covary, indicating that they do not constrain each other evolutionarily. This suggests that female–female aggression and hissing behaviour, despite both being employed in a nest defence context, are distinct behavioural strategies, as the former is primarily a form of intraspecific competition while the latter is primarily a form of anti-predation, emphasizing the importance of considering the context in which these behaviours are expressed. Future research should incorporate longitudinal data of individual birds throughout multiple breeding stages and years to investigate the consistency of female–female aggression across breeding stages and in the long term, as well as study the long-term repeatability of hissing behaviour. By doing so, we will gain a more comprehensive understanding of the dynamics of both aggression and hissing behaviour over extended periods.

### Supplementary Information

Supplementary Information.

## Data Availability

The datasets analysed during the current study are available from the corresponding author (Robin van Iersel) on reasonable request.
