# Supplementary material for: Untangling behaviours: independent expressions of female–female aggression and snake-like hissing in the blue tit (Cyanistes caeruleus)
Source: Sci Rep. 2023 Sep 28;13:16346. doi: 10.1038/s41598-023-43652-3 (PMC10539291; doi:10.1038/s41598-023-43652-3)
Supplement: Supplementary file 1 — Supplementary Information. [file 41598_2023_43652_MOESM1_ESM.pdf]

Supplementary materials

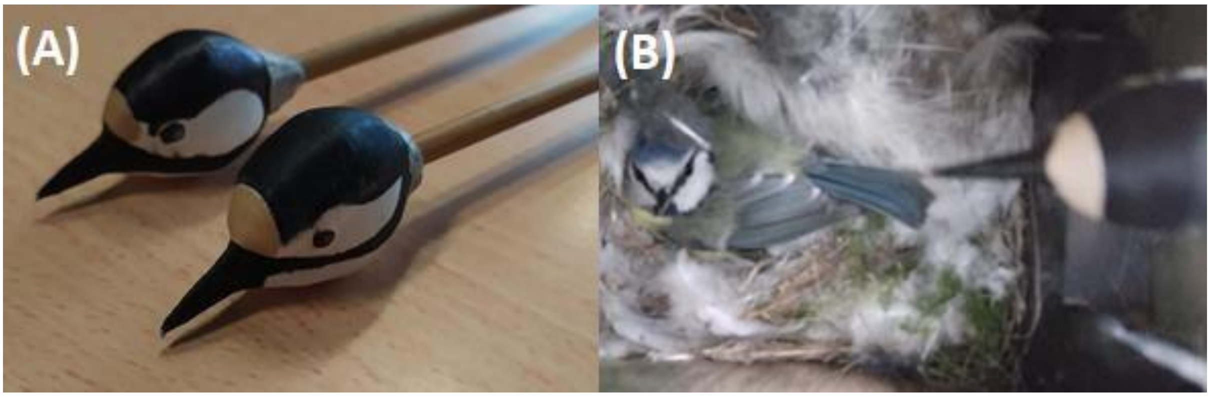

Figure S1: The hissing models used for the hissing tests. Left (A), are the two models used, right (B) a hissing test is being performed.

Table S1: Univariate mixed models for repeatability for each individual aggression parameter. Given are fixed effect estimates and confidence intervals. Fixed effects which do not overlap with zero (95% CI) are in bold.

	Time on decoy	# Pecks	Time in nest box	Time in entrance
Fixed effects	β [CI]	β [CI]	β [CI]	β [CI]
Intercept	**77.99 [29.67, 121.61]**	**1.64 [0.81, 2.46]**	**135.11 [64.70, 195.50]**	0.74 [-0.93, 2.12]
Age – Older	25.61 [-18.21, 69.98]	0.23 [-0.67, 1.24]	**-76.68 [-136.04, -12.36]**	-0.11 [-1.67, 1.61]
Clutch size	6.07 [-12.19, 23.23]	0.07 [-0.28, 0.44]	8.31 [-16.20, 32.64]	**0.73 [0.14, 1.32]**
Julian Date	-10.57 [-36.77, 15.90]	-0.17 [-0.61, 0.35]	6.63 [-22.10, 39.37]	**-1.36 [-2.37, -0.43]**
Decoy B	29.58 [-7.45, 69.33]	-0.03 [-0.86, 0.79]	-17.63 [-73.66, 42.41]	0.12 [-1.16, 1.26]
Decoy C	1.42 [-36.65, 45.73]	-0.20 [-1.07, 0.60]	-0.71 [-55.54, 58.40]	-0.35 [-1.57, 0.91]
Start time	9.39 [-6.06, 27.73]	0.07 [-0.29, 0.45]	-7.11 [-30.07, 17.24]	0.39 [-0.15, 0.91]
Observer B	-12.61 [-54.74, 31.09]	0.50 [-0.34, 1.27]	39.96 [-22.45, 98.79]	0.15 [-1.16, 1.35]
Observer C	-8.30 [-51.31, 33.00]	0.07 [-0.83, 0.97]	35.93 [-23.94, 92.83]	0.35 [-1.00, 1.55]
